# Data for Decision-Making: Exploring the Division of Nutrition, Physical Activity, and Obesity’s Data, Trends, and Maps

**DOI:** 10.5888/pcd16.190043

**Published:** 2019-09-26

**Authors:** Samantha J. Lange, Latetia V. Moore, Deborah A. Galuska

**Affiliations:** 1 Oak Ridge Institute for Science and Education, Research Participation Program, Division of Nutrition, Physical Activity, and Obesity, Centers for Disease Control and Prevention, Atlanta, Georgia; 2 Division of Nutrition, Physical Activity, and Obesity, National Center for Chronic Disease Prevention and Health Promotion, Centers for Disease Control and Prevention, Atlanta, Georgia

## Abstract

Public health practitioners need quick and easy access to reliable surveillance data to monitor states’ progress over time, compare benchmarks nationally or among states, and make strategic decisions about priorities and resources. Data, Trends, and Maps (DTM) at https://www.cdc.gov/nccdphp/dnpao/data-trends-maps/index.html is a free, online interactive database that houses and displays data on nutrition, physical activity, breastfeeding, and obesity that practitioners can use for public health action. Created in 2015 by the Centers for Disease Control and Prevention’s (CDC) Division of Nutrition, Physical Activity, and Obesity, DTM was updated and relaunched in April 2017 with the capability to customize and download data sets directly; DTM also has other user-friendly features, such as visualization options. Since its relaunch, DTM has received more than 386,000 page views from approximately 110,000 unique visitors. However, the potential exists for more widespread use of DTM if more public health practitioners understood what the site offered and how others have used it in the field. Here, we explain how public health practitioners can explore the most recent state-level data on nutrition, physical activity, breastfeeding, and obesity and use this data to inform programmatic and policy efforts to prevent and control chronic diseases. We demonstrate 3 different ways practitioners can visualize data (ie, Explore by Location, Explore by Topic, and the Open Data Portal) and present 3 real-world examples to highlight DTM’s utility as a public health tool.

**Summary**

**What is already known on this topic?**

The Centers for Disease Control and Prevention’s (CDC) Data, Trends, and Maps (DTM) (https://www.cdc.gov/nccdphp/dnpao/data-trends-maps/index.html) is an online, interactive database for state-specific data on nutrition, physical activity, breastfeeding, and obesity. DTM compiles information from several surveillance systems into one free, easily-accessible platform.

**What are the implications for public health practice?**

DTM connects state-level public health practitioners, policy makers, and other users to the reliable surveillance data they need.

**What is added by this report?**

Three case studies illustrate how practitioners have used DTM to supplement grant funding applications, support collaboration with partners, and facilitate data-based decision-making. By understanding DTM’s content and user-friendly capabilities, more practitioners can use data to support effective public health action.

## The Cornerstone of Public Health Practice

According to the World Health Organization, public health surveillance is “the continuous, systematic collection, analysis, and interpretation of health-related data needed for the planning, implementation, and evaluation of public health practice” (1). As a core public health function, surveillance allows us to track long-term trends, identify critical problems and populations most at risk, determine priorities for program and policy implementation, and monitor progress toward goals.

Surveillance of nutrition, physical activity, and obesity is particularly important because of their strong associations with leading causes of preventable death, including cardiovascular disease, type 2 diabetes, and many cancers (2–4). Obesity affects more than 93 million adults and 13 million children and adolescents, approximately 40% and 19% of the US adult and youth populations, respectively (5). Furthermore, many Americans are not physically active enough and they consume unhealthy diets (6–8). For example, less than one-quarter of US adults meet both the aerobic and muscle strengthening physical activity guidelines (6); most do not eat enough fruits and vegetables (7), and they consume too many added sugars, salt, and saturated fats (8). Environments where people live, learn, and work can also support or hinder their ability to consume affordable, healthful foods, engage in regular physical activity, and achieve and maintain a healthy weight (9,10). Thus, surveillance of policy and environmental supports is crucial, as it helps with understanding the context for behaviors.

In the United States, several surveillance systems monitor dietary patterns, physical activity, breastfeeding, and obesity at the state level. The Behavioral Risk Factor Surveillance System (BRFSS), Youth Risk Behavior Surveillance System (YRBSS), National Immunization Survey (NIS), and Women, Infants, and Children Participant and Program Characteristics (WICPC) are a few notable examples (11–14). However, these data are housed on multiple websites, and statistical summaries of the data are in multiple reports. In addition, information regarding state policy and environmental supports for these behaviors is not easily accessible.

To help state-level public health practitioners efficiently find needed data for planning and decision-making, the Centers for Disease Control and Prevention’s (CDC) Division of Nutrition, Physical Activity, and Obesity (DNPAO) created Data, Trends, and Maps (DTM) in 2015. Other potential users of the site include state policy makers, researchers, journalists, and grant writers. To better serve users, we updated DTM in 2017 with the capability to customize and directly download data sets plus a wider range of visualization options. Here, we demonstrate how DTM can be used to explore state-level data on nutrition, physical activity, breastfeeding, and obesity and inform programmatic and policy efforts to promote healthy behaviors and prevent chronic diseases. We describe the content and interactive features of DTM, illustrate 3 ways users can visualize data on the site, and highlight DTM’s usefulness as a tool to support planning and decision-making through 3 real-life examples.

## One-Stop Shop for State-Level Data

DTM is an online, interactive database for state-specific data on nutrition, physical activity, breastfeeding, and obesity. DTM aggregates information from several ongoing state-based surveillance systems, including BRFSS, YRBSS, NIS, WICPC, and various policy and environmental data sources, and presents the data on a single platform. In this way, DTM serves as a bridge between ongoing data collection and public health practice, efficiently connecting users to the surveillance information they need. The database currently houses 56 indicators in 6 priority topic areas: obesity and weight status, physical activity, fruits and vegetables, breastfeeding, sugary drinks, and television viewing (Table). DTM indicators were originally selected to align with DNPAO’s programmatic priorities and to support grantees that work in priority topic areas. Some indicators quantify health behaviors, such as breastfeeding and fruit and vegetable consumption. Other indicators describe state-level environmental or policy supports for healthy eating, active living, and obesity prevention, such as the presence of a Complete Streets policy and whether child care regulations meet national standards for avoiding sugar. The combination of both behavioral and policy or environmental indicators gives public health practitioners and other DTM users a context for understanding the multifactorial nature of chronic diseases.

**Table:** Behavior and Environmental or Policy Indicators by Topic Area, Data, Trends, and Maps, 2019

Topic Area	Category	Indicator	Years Available	Data Source
**Obesity and Weight Status**	Behavior	Adults who have obesity	2011–2017, annually	BRFSS
		Adults who have an overweight classification	2011–2017, annually	BRFSS
		Adolescents who have obesity	2001–2017, every other year	YRBSS
		Adolescents who have an overweight classification	2001–2017, every other year	YRBSS
		WIC 2–4 year olds who have an overweight classification	2008, 2010, 2012, 2014	WICPC
		WIC 2–4 year olds who have obesity	2008, 2010, 2012, 2014	WICPC
		WIC 3–23 month olds who have a high weight–for–length	2008, 2010, 2012, 2014	WICPC
**Physical Activity**	Behavior	Adults who usually walk or bike to work	2006–2010, 2011–2015^a^	American Community Survey
		Adults aerobically active 150 minutes	2011, 2013, 2015, 2017	BRFSS
		Adults meeting aerobic and muscle strengthening guidelines	2011, 2013, 2015, 2017	BRFSS
		Adults aerobically active 300 minutes	2011, 2013, 2015, 2017	BRFSS
		Adults meeting muscle strengthening guidelines	2011, 2013, 2015, 2017	BRFSS
		Adults who engage in no leisure–time physical activity	2011–2017, annually	BRFSS
		Adolescents who are physically active daily	2001–2017, every other year	YRBSS
		Adolescents who participate in daily physical education	2001–2017, every other year	YRBSS
**Physical Activity**	Environmental or Policy	Adults living within .5 mile of at least one park	2015	National Environmental Public Health Tracking Network
		Youth with parks/rec centers/sidewalks in their neighborhoods	2016	National Survey of Children’s Health
		State requires physical activity for child care (preschool)	2010–2016, annually	NRC report
		State has adopted some form of a Complete Streets policy	2012–2016, annually	National Complete Streets Coalition
		State guidance on policy for joint use of school facilities	2012	SHPPS
		State guidance on policies for school recess	2012	SHPPS
		State guidance on policies for physical activity in PE class	2012	SHPPS
		State guidance on policies for walking/biking to/from school	2012	SHPPS
**Fruits and Vegetables**	Behavior	Adults who consume fruit <1 time daily	2017	BRFSS
		Adults who consume vegetables <1 time daily	2017	BRFSS
		Adolescents who consume fruit <1 time daily	2001–2017, every other year	YRBSS
		Adolescents who consume vegetables <1 time daily	2001–2017, every other year	YRBSS
**Fruits and Vegetables**	Environmental or Policy	State-level Food Policy Council	2018	SIR on Fruits and Vegetables
		Farmers markets per 100,000 residents	2009, 2012, 2017	SIR on Fruits and Vegetables
		Number of food hubs in each state	2012, 2017	SIR on Fruits and Vegetables
		Number of Local Food Policy Councils	2018	SIR on Fruits and Vegetables
		Farmers markets that accept SNAP benefits	2012	SIR on Fruits and Vegetables
		Farmers markets that accept WIC coupons	2012, 2017	SIR on Fruits and Vegetables
		State child care regulations meet national standards for serving fruits	2010–2017, annually	NRC report
		State child care regulations meet national standards for serving vegetables	2010–2017, annually	NRC report
		State-level farm to school/preschool policy	2011	SIR on Fruits and Vegetables
		Secondary schools that offer a self-serve salad bar	2014, 2016	School Health Profiles
**Breastfeeding**	Behavior	Breastfed infants supplemented with formula within 2 days	2004–2015, annually	NIS
		Breastfed infants supplemented with formula before 3 months	2004–2015, annually	NIS
		Breastfed infants supplemented with formula before 6 months	2004–2015, annually	NIS
		Infants ever breastfed	2000–2015, annually	NIS
		Infants breastfed at 6 months	2000–2015, annually	NIS
		Infants breastfed at 12 months	2000–2015, annually	NIS
		Infants exclusively breastfed through 3 months	2004–2015, annually	NIS
		Infants exclusively breastfed through 6 months	2004–2015, annually	NIS
**Breastfeeding**	Environmental or Policy	Maternity Practice in Infant Nutrition and Care (mPINC) score	2007– 2015, every other year	mPINC Survey
		Number of International Board Certified Lactation Consultants (IBCLCs) per 1,000 live births	2007–2016, annually	Breastfeeding Report Card
		Number of La Leche League leaders per 1,000 live births	2011–2016, annually	Breastfeeding Report Card
		Births occurring at designated "baby friendly" hospitals	2007–2018, annually	Breastfeeding Report Card
**Sugary Drinks**	Behavior	Adolescents who drank soda daily	2007–2017, every other year	YRBSS
**Sugary Drinks**	Environmental or Policy	Schools that allowed students to purchase soda pop or fruit drinks	2010, 2012, 2014, 2016	School Health Profiles
		Schools that allowed students to purchase sports drinks	2010, 2012, 2014, 2016	School Health Profiles
		State child care regulations meet national standards for avoiding sugar	2010–2016, annually	NRC report
**Television Viewing**	Behavior	Adolescents watching 3 or more hours of TV daily	2001–2017, every other year	YRBSS
**Television Viewing**	Environmental or Policy	State child care regulations meet national standards for media for children under 2	2010–2016, annually	NRC report
		State child care regulations meet national standards for media for children 2 and older	2010–2016, annually	NRC report

Abbreviations: BRFSS, Behavioral Risk Factor Surveillance System; DTM, Data, Trends, and Maps; NRC, National Resource Center for Health and Safety in Child Care and Early Education; NIS, National Immunization Survey; PE, physical education; SHPPS, School Health Policies and Practices Study; SIR, State Indicator Report; SNAP, Supplemental Nutrition Assistance Program; WIC, Special Supplemental Nutrition Program for Women, Infants, and Children; WICPC, Women, Infants, and Children Participant and Program Characteristics; YRBSS, Youth Risk Behavior Surveillance System.

a 5-year estimates.

Other key characteristics of DTM include being up-to-date, reliable, and easily accessible. Approximately 3 to 4 times per year, DNPAO updates DTM to ensure availability of the most recent information on the indicators. Yet data from previous years remain on the site so that users can view trends over time. Before uploading new data, database managers perform quality assurance checks to ensure reliability and accuracy of data and visualizations. Releases are promoted through social media to more than 45,000 Facebook and Twitter followers and to partners and grantees through email distribution lists. Furthermore, DTM’s web-based platform is free to access and supports both mobile and desktop viewing, which adds to the site’s usability by a variety of audiences.

## Exploring Data, Trends, and Maps

To meet the diverse needs of public health practitioners and other DTM users, data can be obtained and visualized using 3 methods: Explore by Location, Explore by Topic, and the Open Data Portal.

**Figure 1:**
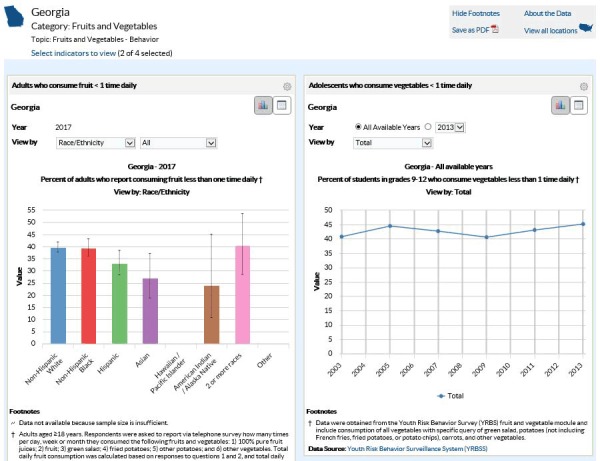
**Screenshot Example of the Explore by Location visualization Feature in the Data, Trends, and Maps database at** https://www.cdc.gov/nccdphp/dnpao/data-trends-maps/index.html **.**

*Explore by Location*: By using DTM’s Explore by Location feature, users can see all available indicators for a topic in a particular US state or territory. DTM presents the indicators for the selected locale in side-by-side panels, displaying up to 8 indicators simultaneously. This method of data exploration is the most commonly used, comprising approximately 80% of overall site traffic. In Explore by Location, users can customize how they visualize the data based on their needs. This might include viewing data by demographic characteristics such as race/ethnicity or age group to identify disparities, charting indicators over time to monitor progress, or saving graphs for presentations or other purposes. Figure 1 contains an example.

Steps to create the visualization shown in Figure 1 are 1) Select your state of interest from the map on the DTM home page; 2) Select your desired topic area, such as Fruits and Vegetables-Behavior; and 3a) Click the View By drop-down menu to display the data by demographics, such as race/ethnicity, or 3b) Click All Available Years to display the data trend over time.

**Figure 2:**
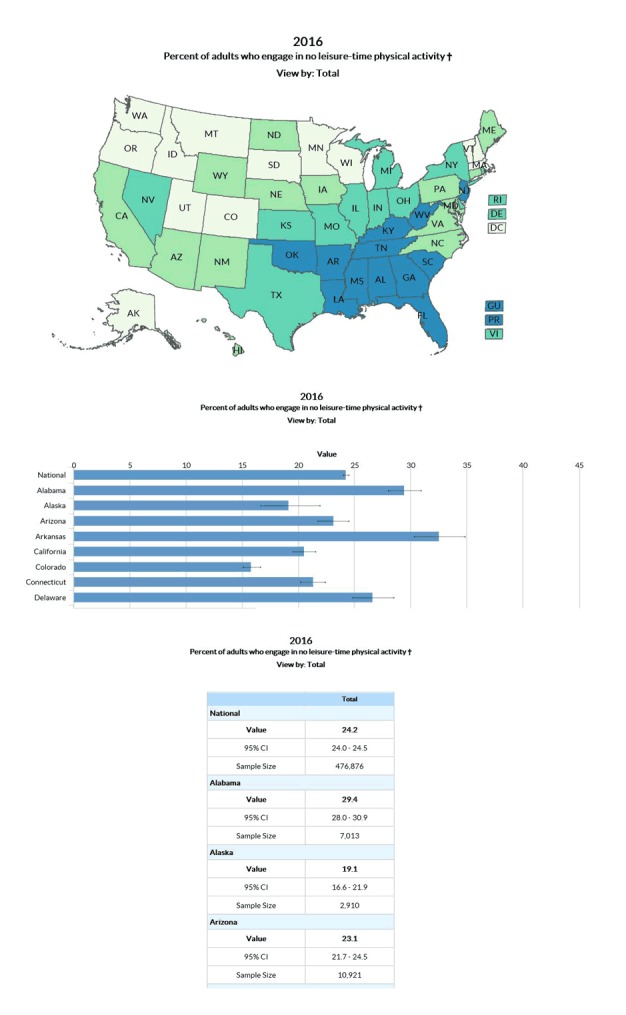
Three Ways Users Can View Data for a Specific Indicator for all Available Locations via Explore by Topic on Data, Trends, and Maps: US map, bar chart, and data table.

*Explore by Topic*: The Explore by Topic feature displays 1 indicator at a time for all US states and territories with available data. This feature allows users to compare a health indicator across states or compare a benchmark to available national estimates (Figure 2). For example, users viewing a map of adults who do not engage in leisure-time physical activity can click on various states to obtain state-specific percentages or quickly compare them using the map’s color-coded legend. Additionally, users can select the chart view to see a bar graph for all states and the national total. Selecting the table view will display a list of state-specific estimates for the indicator of interest. Like Explore by Location, Explore by Topic allows users to explore differences in indicators by demographics, view previous years of data, and save visualizations.

Steps to create the visualization shown in Figure 2 are 1) Select your desired topic area, such as Physical Activity-Behavior, from the DTM home page; 2) Select the specific indicator you are interested in, such as Percent of adults who engage in no leisure-time physical activity; and 3) Use the icons at the top right of the map to view the data in a bar chart or table.

*Data Portal*: Lastly, the Open Data Portal connects users directly to aggregate data estimates by state (eg, the number of food hubs in Washington, DC) and demographics (eg, the prevalence of obesity among Hispanics in the United States) from the various surveillance systems that contribute to DTM. In the Open Data Portal, users can browse, filter, and download data sets to complete their own analyses. They can also view ready-made figures for individual indicators, such as the percent of adults who engage in no leisure-time physical activity by demographic characteristics; customize their own graphics; and find additional data set details, such as contact information for inquiries or technical assistance. To access the Data Portal from the DTM home page, users should click the link that reads “Nutrition, Physical Activity, and Obesity Data Portal.”

## DTM in Action

### Use of Data, Trends, and Maps

State public health practitioners can use DTM for multiple data-driven purposes. They can contextualize a health problem by exploring the prevalence of behavioral risk factors and the environmental supports related to reducing those risk factors in their state. Practitioners can explore data to monitor progress over time, compare their data to national benchmarks or to other states, and guide decision-making processes for effective public health action. Decisions might include how to maximize limited resources by prioritizing and targeting health disparities for the most effective intervention within diverse communities and populations.

Since the launch of the updated site in April 2017, DTM has received more than 386,000 page views from approximately 110,000 unique visitors with more activity occurring when new data are added. Ninety-eight percent of DTM users are from outside of CDC. Although we know that thousands of people use DTM, we have not systematically evaluated how people are using the data. Even more state public health practitioners and other users might benefit from DTM if they understand how it has been used by others. To illustrate the usefulness of DTM, we provide 3 case examples of how external users — public health teams in Florida, North Carolina, and Rhode Island — have used DTM in the field to support effective public health actions.

### Example Case 1: Prioritization and Grant Funding

The Community and Clinical Connections for Prevention and Health Branch (CCCPH) at the North Carolina Division of Public Health uses DTM extensively to contextualize the burden of nutrition and physical activity risk behaviors among North Carolina residents and recognizes opportunities for policy implementation (personal communication, August 2018). To accomplish this, state evaluators often use DTM’s Open Data Portal to export relevant data sets for their own analyses. Evaluators also explore maps and tables on the site to compare North Carolina’s performance to national estimates and ranks among other states.

The branch’s lead evaluator at CCCPH commented, “DTM provides a useful and easy-to-navigate array of physical activity and nutrition data. It helps us identify physical activity and nutrition issues that our state is doing well in and issues we need to prioritize for improvement.” He also noted that the breadth of DTM’s policy and environmental data are particularly valuable because those types of indicators are not as readily available to practitioners as behavioral ones. With this information, the evaluation team better understands the extent of critical health issues and can accurately document priorities in funding applications and report performance measures in health impact statements. Recently, CCCPH successfully applied for a federal grant to implement CDC’s State Physical Activity and Nutrition Program. Using state and national data from DTM, the branch highlighted the lack of physical activity supports for North Carolinians, including that the state ranks 47^th^ in the nation for the percent of adults living within one-half mile of a park (13.5%), compared with the national estimate of 37.3%. CCCPH was also able to identify opportunities to increase physical activity in places where people live, learn, and work.

### Example Case 2: Programs and Partnerships

The Epidemiology and Evaluation Team at the Florida Bureau of Chronic Disease Prevention uses DTM to support their programmatic and partnership work in breastfeeding (personal communication, July 2018). The acting program chief observed that her team primarily uses DTM’s Explore by Location feature to examine breastfeeding exclusivity and duration behaviors, both over time to track progress and by demographics, such as race/ethnicity and educational attainment, to identify disparities and populations at highest risk. She has also examined DTM’s policy and environmental indicators related to breastfeeding, particularly “the percent of live births occurring at facilities designated as ‘baby-friendly’” at the state and national levels (15). Baby-friendly facilities are “centers of support where evidenced-based care is provided, education is free from commercial interests, all infant feeding options are possible, and individual preferences are respected” (15). Florida’s Epidemiology and Evaluation Team, in partnership with the Florida Breastfeeding Coalition and the Florida Hospital Association, conducted a statewide webinar to highlight August as Breastfeeding Awareness Month. The purpose of the webinar was to raise awareness of hospital maternal care practices that support breastfeeding and recognition opportunities and to boost participation in Florida’s Baby Steps to Baby Friendly Hospital initiative. In preparation, the team used DTM’s Export CSV (comma-separated values) file function to extract relevant data from online tables and designed visuals to match partner-branded presentation materials. CSV is a type of file format that is used to store tabular data, such as in a spreadsheet or database. CSV formatted files can be imported to and exported from programs that store data in tables.

### Example Case 3: Informed Decision-Making

The Physical Activity and Nutrition Program (PAN) at the Rhode Island Department of Health uses DTM to support data-based decision-making. For example, PAN staff examined DTM indicators by income status and other social determinants of health to identify groups that were disproportionately affected by obesity and its leading risk factors, such as no leisure-time physical activity. Identifying target populations enabled PAN to deliver resources where they were needed most and to yield the greatest public health impact. A PAN program evaluator remarked that DTM is particularly helpful to her team because it “reduces the burden on departmental epidemiologists and empowers other staff to make data-informed decisions in obesity prevention” (personal communication, October 2018). This reflects DTM’s intended purpose of being usable by a variety of audiences, not only those who regularly work with data. PAN staff indicated they use DTM to quickly obtain data that they need to answer requests from department leadership, respond to public or stakeholder inquiries, and inform legislation for the state of Rhode Island.

## Limitations of DTM

Despite its strength as a broad database of state-level data on behaviors and environmental supports for nutrition, physical activity, breastfeeding, and obesity, DTM has some limitations. First, DTM was initially developed to serve the programmatic needs of DNPAO’s state grantees; thus, it does not provide local-level data or an exhaustive list of relevant policy indicators. However, other nutrition and physical activity data sources contain this information, including BRFSS 500 Cities (16) and the US Department of Agriculture’s Food Atlas (17), and can supplement information from DTM. Second, although DTM receives substantial traffic, it might not reach all types of users who could benefit from it, and we are unable to determine exactly who DTM users are. We have, however, worked to ensure that DTM is accessible to a variety of potential users. For example, we have conducted how-to presentations for DNPAO program staff who work directly with state practitioners and grant awardees to facilitate DTM use. Third, there is a natural delay between when data are collected by BRFSS and other surveillance systems, when they are publicly available for analysis, and when they are added to DTM, but we try to minimize delays by analyzing and updating the database 3–4 times per year.

## Conclusion

DTM, at https://www.cdc.gov/nccdphp/dnpao/data-trends-maps/index.html, is a free, online, interactive database for state-specific data on nutrition, physical activity, breastfeeding, and obesity. To provide state public health practitioners with the data they need for planning, tracking, and decision-making, DTM contains information from multiple surveillance systems on one easily accessible platform. Public health practitioners and other users can visualize this information using 3 different methods — Explore by Location, Explore by Topic, and the Open Data Portal — or customize and download data sets directly from the site. Practitioners use DTM for a variety of purposes from contextualizing health problems in their states to applying for grant funding and working with partner organizations. By understanding DTM’s content and user-friendly capabilities, additional practitioners, policy makers, and other users can employ DTM to support effective public health practice.
